# Physical activity, overweight and central adiposity in Swedish children and adolescents: the European Youth Heart Study

**DOI:** 10.1186/1479-5868-4-61

**Published:** 2007-11-19

**Authors:** Francisco B Ortega, Jonatan R Ruiz, Michael Sjöström

**Affiliations:** 1 Unit for Preventive Nutrition, Department of Biosciences and Nutrition, Karolinska Institutet, Huddinge, Sweden; 2 The EFFECTS-262 research group, Department of Physiology, University of Granada, Granada, Spain

## Abstract

**Background:**

The aim of this work was to study the associations of physical activity (PA) and other factors predisposing to overweight, with overweight and central adiposity in children and adolescents.

**Methods:**

A total of 557 Swedish children (9.5 ± 0.3 y) and 517 adolescents (15.6 ± 0.4 y) from the European Youth Heart Study participated in this study. Logistic regression analyses were used to examine the associations of PA, as measured by accelerometry, and other determinants (i.e. television viewing, birth weight, maternal educational level and parental overweight) with total and central adiposity. Body mass index and waist circumference cut-off values proposed by the IOTF and the Bogalusa Heart Study (i.e. waist measures for predicting risk factors clustering, hereinafter called high-risk waist circumference), respectively, were used. Fatness was estimated from skinfold thicknesses and dichotomized using the 85^th ^sex- and age-specific percentile (high/low).

**Results:**

Children and adolescents who had a low level (first tertile) of vigorous PA, were more likely to be overweight (including obesity) and to have a high-risk waist circumference, than those with a high level (third tertile) of vigorous PA. Similarly, those subjects who had a low or middle level (second tertile) of total PA were more likely to be overweight than those who had a high level of total PA. Among the PA variables, only vigorous PA was associated with high total fatness. Birth weight and television viewing, were also associated with higher odds of having a high-risk waist circumference, but these associations were attenuated once either total or vigorous PA variable was included in the model. Those subjects who had two overweight parents were more likely to be overweight and to have a high-risk waist circumference independently of PA variables, compared to those whose parents were not overweight.

**Conclusion:**

Low levels of total PA and especially vigorous PA may play an important role in the development of overweight and excess of central adiposity in children and adolescents, independently of a number of factors such as television viewing and birth weight. In addition, the data suggest that the association between television viewing and central fat deposition could be attenuated if enough vigorous PA is accumulated. Longitudinal and intervention studies are needed to confirm these findings.

## Background

The increase in childhood overweight and obesity is a public health concern in developed countries [1-4]. Central obesity may be a better predictor than overall obesity for the risk of cardiovascular disease and type II diabetes, and in adults it seems to be a strong predictor of morbidity and mortality independent of body mass index (BMI) [1,4]. Most metabolic disturbances associated with central adiposity, such as undesirable lipid and insulin concentrations, show their onset during childhood [5]. Therefore, the study of central adiposity at these ages and its relationships with modifiable factors that may influence it is important from a health promotion perspective. In this context, a better understanding of the relative role of physical activity (PA) and other determinant factors for the development of total and central overweight/obesity at young ages is needed. Other factors such as time spent in television (TV) viewing [6], a high or low birth weight [7,8], the level of maternal education [9,10] and parental overweight [11] seem to be associated with the adiposity level in young people, and require further research.

The relationship of levels and patterns of PA with total and central adiposity in children and adolescents is unclear [12-18], which may be due to the different methodologies used to measure PA. Studies using objective methods to assess levels and patterns of PA have recently been published [19-21]. However, a better understanding of the association between PA and overweight and abdominal adiposity is still required. Since childhood obesity in a complex pathological condition that is affected by multiple variables, the analysis of a number of established determinant factors when aiming to study the potential effect of one specific factor on adiposity (e.g. PA) is fundamental. In this regard, comprehensive insights are needed on the associations between objectively measured PA variables and overweight and central adiposity, accounting for several proposed determinant factors, such as TV viewing, birth weight, maternal educational level and parental overweight in young people.

The aim of this report was to study the independent associations of objectively measured PA and other factors predisposing to overweight, with overweight and central adiposity in children and adolescents.

## Methods

### Study design

A total of 557 children (9.5 ± 0.3 y) and 517 adolescents (15.6 ± 0.4 y) from the Swedish part of the European Youth Heart Study (EYHS) [22] with valid data for waist circumference and BMI and were included in this study. The 97% of these subjects had complete and valid data for sexual maturation status, 94% for TV viewing, 90% for birth weight, 88% for maternal educational level, 88% for parental overweight and 71% for objectively measured PA.

Data collection took place from September 1998 to May 1999 in 37 schools from eight municipalities (Botkyrka, Haninge, Huddinge, Nynäshamn, Salem, Södertälje, Tyresö, and Örebro) in Sweden. Study design, sampling procedures, selection criteria, participation rates and study protocol have been reported elsewhere [23].

The study was approved by the Research Ethics Committees of Örebro County Council and Huddinge University Hospital. One parent or legal guardian provided written informed consent, and all children and adolescents gave verbal consent.

### Objectively measured physical activity

Physical activity was measured with an activity monitor (MTI model WAM 7164, Manufacturing Technology Inc., Shalimar, Florida, formerly known as Computer Science and Applications Inc.) worn at the right hip. Accelerometers provide a measure of frequency, intensity, and duration of movement, allowing data to be analyzed over user-defined intervals (epochs). In this study, the epoch duration was set at 1 min, since this epoch duration is widely used in field-based studies [21,24], allowing data to be collected for sufficiently long time.

The participants were asked to wear the accelerometer over four consecutive days (including necessarily both weekdays and weekend days) and the inclusion criteria were set as: at least three days of recording and with a minimum of 10 hours registration per day.

Total PA was expressed as total counts recorded, divided by total daily registered time (counts/min). The time spent in moderate and vigorous PA was calculated and presented as the average time per day during the complete registration (min/d). Moderate PA (3–6 metabolic equivalents, METs) and vigorous PA (> 6 METs) intensities were defined upon cut-off limits published elsewhere [25]. The cut-off limits used in this study have been shown to be valid for estimating time spent in moderate and vigorous PA in children and adolescents [26]. The time spent in at least moderate intensity level (> 3 METs) was calculated as the sum of time spent in moderate and vigorous PA (MVPA, min/day). The proportion of subjects who met the current PA recommendations of 60 min or more of MVPA daily was then calculated [27].

### Other determinant factors

The time spent in TV viewing was self-reported by the subjects by means of the question "How many hours of TV do you usually watch?" The answer was classed as either ≤ 2 h/day or > 2 h/day [6]. Parents reported the subjects' birth weights, as well as their own height and weight. Birth weight was categorized as < 2500 g, between 2500 and 4000 g or > 4000 g [28]. The socioeconomic status was defined by the maternal educational status as below university education and university education [9,10]. The BMI for parents was calculated and the overweight status according to international cut-off for adults was determined (≥ 25 Kg/m^2 ^= overweight, and ≥ 30 Kg/m^2 ^= obesity). The validity of BMI based on self-reported weight and height in adults has been documented elsewhere [29].

### Physical examination and definitions

Commonly used markers for overweight and adiposity are BMI and waist circumference. Body mass index is a reasonable proxy for total obesity when used with cut-off values adapted to each age and sex [30], while waist circumference is used to express central adiposity [31,32].

Height and weight of children were measured by standardized procedures. Body mass index was calculated as weight divided by height squared (Kg/m^2^). The age- and sex-specific BMI cut-off values proposed by the International Obesity Task Force [30], were used to categorize the subjects as non-overweight, overweight and obese. For the analyses, this variable was dichotomized as non-overweight and overweight, which includes obesity (hereinafter called overweight).

Waist circumference was measured with a metal anthropometric tape midway between the lower rib margin and the iliac crest, at the end of a gentle expiration. The age- and sex-specific waist circumference cut-off values for predicting risk factor clustering proposed by the Bogalusa Heart Study were used to class the individuals as having a high or low metabolic risk (hereinafter called high/low-risk waist circumference) [33]. Several percentile-based reference values for central obesity have been published elsewhere [31,34], however the sex- and age-specific cut-off values chosen for this study provide meaningful information about a waist circumference size associated with higher metabolic risk, and not only a high level of abdominal fatness.

Skinfold thicknesses were measured with a Harpenden caliper (Baty International, Burguess Hill, U.K.) on the left side of the body according to the criteria described by Lohman et al. [35]. All measurements were taken twice and in rotation, and the mean value was calculated. If the difference between the two measurements was more than two millimeters a third measurement was taken and the two closest measurements were averaged. The Slaughter's equations [36] have been proposed as the most accurate equations for the estimation of body fat percentage from skinfold thickness in young people [37]. Consequently, these equations were used to calculate the percentage of body fat in this study. According to previous research [38], being above the sex- and age-specific 85^th ^centile of body fat percentage was used as a cut-off value for defining individuals with high levels of total adiposity (hereinafter called high total fatness). Pediatric overweight is internationally defined by sex- and age-specific cut-offs for BMI [30]. We have used BMI as the main outcome for assessing total overweight, since international standards for body fat percentage in young people, estimated from skinfold thickness, have not been established yet. Nevertheless, whether the results differ when body fat percentage is used instead of BMI was also studied.

Pubertal stage was assessed by a trained researcher, after a brief observation, according to Tanner and Whitehouse [39].

### Statistical analysis

Study sample characteristics are presented as means and standard deviations (SD), unless otherwise stated. The effects of sex and age on the sample characteristics (continuous variables) were analysed by analysis of variance (two-way ANOVA), with sex and age as fixed factors, and the sample characteristics as dependent variables. All the residuals showed a satisfactory pattern. Nominal data were analysed by Chi-square tests, for sex and age groups consecutively.

The association of PA variables and the other determinant factors with BMI (non-overweight/overweight) and waist circumference (low/high-risk waist circumference) was analyzed by binary logistic regression.

No significant interaction was found between sex and age in the associations between the predictors and outcome variables. Therefore, the analysis was performed for the whole sample together, entering sex and age into the models as covariates.

Total, moderate and vigorous PA variables were recoded into tertiles within each sex and age (low, middle and high PA levels, corresponding to the first, second and third tertile, respectively). Since most of the children met the current PA recommendations (≥ 60 min/day of MVPA), the analysis for this dichotomized variable was only performed for the adolescent group.

For the predictors for which significant associations were observed, the analysis was redone after controlling for the rest of the significant predictors, in order to examine whether the associations were independent of each other. In addition, the analyses were performed using percentage body fat (> 85^th ^sex- and age-specific percentile) instead of BMI, to examine whether the results changed when different indices for assessing total overweight were used.

The outcome did not change when the analyses were performed entering the PA variables as continuous variables instead of as categorical (tertiles). Categorized variables were finally used to allow a more simple and meaningful interpretation. The analysis was performed using SPSS v.15.0 software for Windows. For all analyses, the significance level was 5%.

## Results

Physical characteristics and PA variables are shown in Table 1. Data on TV viewing, birth weight, maternal educational level and parental overweight are displayed in Table 2. In all PA variables studied, boys were more active than girls and children more active than adolescents. Regarding meeting PA recommendations, the differences between boys and girls were greater in adolescents than in children. Nearly all the children met the PA recommendations, whereas 71% of the adolescents boys, and 60% of the adolescents girls did not meet the PA recommendations.

**Table 1:** Characteristics of the study subjects by sex and age group.

	Children		Adolescents	
	
	Boys (n = 269)	Girls (n = 288)	Boys (n = 238)	Girls (n = 279)
Age (y)	9.5 ± 0.3	9.5 ± 0.4	15.6 ± 0.4	15.5 ± 0.4
Sexual maturation status (%): *†				
Pre-pubescents (Stage 1)	98.4	57.6	0.5	0
Pubescents (Stages 2, 3 and 4)	1.6	42.4	16.3	45.7
Post-pubescents (Stage 5)	0	0	83.3	54.3
Weight (Kg) *†	33.4 ± 6.2	33.7 ± 6.7	64.2 ± 10.7	57.8 ± 8.8
Height (m) *†	1.4 ± 0.1	1.4 ± 0.1	1.8 ± 0.1	1.6 ± 0.1 21.2 ± 2.7
Body mass index (Kg/m^2^) †	17.2 ± 2.4	17.3 ± 2.4	20.7 ± 2.8	
Overweight/obesity (%) §	13.4	17.7	12.5	12.7
Waist circumference (cm) *†	60.7 ± 6.0	60.2 ± 6.1	73.8 ± 7.1	70.0 ± 6.7
High-risk waist circumference (%) ‡II	27.2	28.6	17.4	30.1
Percentage body fat *†	16.1 ± 6.3	18.6 ± 5.6	14.0 ± 6.5	23.4 ± 5.5
Total amount of PA (counts/min) *†	805 ± 259	665 ± 189	557 ± 199	490 ± 154
Moderate PA (min/d) *†	185 ± 50	159 ± 41	65 ± 31	58 ± 22
Vigorous PA (min/d) *†	35 ± 23	24 ± 15	16 ± 12	11 ± 10
MVPA (min/d) *†	220 ± 65	182 ± 51	81 ± 38	69 ± 28
Meeting PA recommendations:				
≥ 60 min/day of MVPA (%) *†	99.0	99.6	70.1	60.2

Data shown as mean ± standard deviation, unless otherwise stated. PA, physical activity; MVPA, moderate-vigorous PA. Sex differences (*) and age group differences (†) were analysed by Chi-square tests (nominal variables) or two-way ANOVA (continuous variables). ‡ Sex differences were found only in adolescents. §International Obesity Task Force body mass index cut-off values. II Waist circumference cut-off values for predicting risk factor clustering, as proposed by Bogalusa Heart Study

**Table 2:** Television viewing, birth weight, maternal education and parental overweight in Swedish children and adolescents by sex.

	Children		Adolescents	
	
	Boys	Girls	Boys	Girls
TV viewing: > 2 h/day (%) †	23.9	22.5	44.8	40.5
Birth weight (%): *				
< 2.500 g	2.5	5.6	0.9	5.9
2500 g–4000 g	74.0	82.3	77.1	82.2
> 4000 g	23.6	12.0	22.1	11.8
Maternal educational level (%): †				
Below University	68.0	68.1	61.2	55.8
Parental overweight/obesity (%): †‡				
Only father	34.2	37.1	38.9	37.0
Only mother	8.2	15.4	15.9	13.8
Both father and mother	17.7	16.2	19.0	18.5

TV, television. Sex differences (*) and age group differences (†) were analysed by Chi-square tests. ‡ Sex differences were found only in children and age group differences only in boys.

### Objectively measured physical activity

The odds ratios (OR) and confidence intervals (CI) of being overweight and having a high-risk waist circumference according to objectively measured PA variables, after controlling for sex and age, are shown in Table 3. No significant association was found for PA recommendations, moderate PA or MVPA variables.

**Table 3:** Overweight and high-risk waist circumference according to physical activity (PA) variables.

		Overweight *		
		
		OR ‡	95 % CI	*P *≤
Total amount of PA **	High PA level	1	Reference	
	Middle PA level	3.2	1.4–7.4	**0.006**
	Low PA level	2.7	1.2–6.4	**0.019**
Moderate PA **	High PA level	1	Reference	
	Middle PA level	1.7	0.7–3.7	0.218
	Low PA level	2.0	0.9–4.4	0.073
Vigorous PA **	High PA level	1	Reference	
	Middle PA level	1.9	0.8–4.7	0.157
	Low PA level	4.1	1.8–9.5	**0.001**
MVPA **	High PA level	1	Reference	
	Middle PA level	1.0	0.5–2.3	0.976
	Low PA level	2.1	0.9–4.3	0.162
PA recommendations ††	≥ 60 min/day MVPA	1	Reference	
	< 60 min/day MVPA	1.9	0.9–4.3	0.118
		
		High-risk waist circumference †		

Total amount of PA **	High PA level	1	Reference	
	Middle PA level	1.1	0.6–2.1	0.765
	Low PA level	1.0	0.6–2.0	0.913
Moderate PA **	High PA level	1	Reference	
	Middle PA level	1.1	0.6–2.0	0.794
	Low PA level	1.3	0.7–2.4	0.443
Vigorous PA **	High PA level	1	Reference	
	Middle PA level	1.4	0.8–2.7	0.277
	Low PA level	2.1	1.1–3.9	**0.026**
MVPA **	High PA level	1	Reference	
	Middle PA level	0.6	0.3–1.5	0.285
	Low PA level	1.3	0.6–2.8	0.448
PA recommendations ††	≥ 60 min/day MVPA	1	Reference	
	< 60 min/day MVPA	1.4	0.8–.7	0.279

OR, odds ratios; CI, confidence intervals; MVPA, moderate-vigorous PA. * International Obesity Task Force body mass index cut-off values. † Waist circumference cut-off values for predicting risk factor clustering, as proposed by Bogalusa Heart Study. ‡ Logistic regression analysis was performed controlling for sex and age. ** Low, middle and high PA levels represent the 1^st^, 2^nd ^and 3^rd ^tertiles, respectively. They all were age- and sex-specifically calculated. †† Since the number of children not meeting PA recommendations were very small (≤ 1%), this analysis was performed only for adolescents.

Those subjects who had a low (first tertile) or middle level (second tertile) of total PA were more likely to be overweight (*P *= 0.019 and *P *= 0.006, respectively), but not to have a high-risk waist circumference, than those who had a high level (third tertile) of total PA. This association remained significant after controlling for TV viewing, birth weight, and parental overweight (*P *≤ 0.05, data not shown). When sexual maturation status was entered into the model instead of age group, only a trend to the significance was found (for all *P *≤ 0.1, data not shown).

Children and adolescents who had a low level (first tertile) of vigorous PA, had four times higher odds of being overweight (*P *= 0.001) and two times higher odds of having a high-risk waist circumference (*P *= 0.026), compared to those who had a high level (third tertile) of vigorous PA. When these associations were controlled for TV viewing or birth weight, low vigorous PA was still a significant predictor of the odds of being overweight and having a high-risk waist circumference (for all *P *≤ 0.05, data not shown). When controlling for sexual maturation status instead of age groups, the associations between vigorous PA and overweight and high-risk waist circumference remained significant (for all *P *≤ 0.01, data not shown). After controlling for parental overweight, vigorous PA was still associated with the odds of being overweight (*P *≤ 0.05, data not shown).

When high total fatness instead of overweight was entered into the models as dependent variable, only vigorous PA was significantly associated with high total fatness (*P *≤ 0.05, data not shown).

### Other determinant factors

The odds ratios and CI of being overweight and having a high-risk waist circumference according to TV viewing, birth weight, maternal educational level and parental overweight, after controlling for sex and age, are shown in Table 4. Those subjects that spent 2 h/day or more in TV viewing were more likely to have a high-risk waist circumference than those who did not (*P *= 0.004). However, this association did not persist when either total or vigorous PA was entered into the model.

**Table 4:** Overweight and high-risk waist circumference according to several determinant factors.

		Overweight *		
		
		OR ‡	95 % CI	*P *≤
TV viewing time	≤ 2 h	1	Reference	
	> 2 h	1.8	1.0–3.3	0.070
Birth weight	2500–4000 g	1	Reference	
	< 2500 g	1.5	0.4–5.0	0.514
	> 4000 g	1.5	0.7–3.2	0.346
Maternal educational level	University	1	Reference	
	Below University	1.9	1.0–3.6	0.067
Parental overweight	No overweight/obese parents	1	Reference	
	Overweight/obese father	2.0	0.9–4.5	0.111
	Overweight/obese mother	2.8	1.1–7.3	**0.033**
	Both overweight/obese parents	3.4	1.8–4.2	**0.008**
		
		High-risk waist circumference †		

TV viewing time	= 2 h	1	Reference	
	> 2 h	2.1	1.3–3.6	**0.004**
Birth weight	2500–4000 g	1	Reference	
	< 2500 g	0.8	0.2–2.4	0.619
	> 4000 g	2.2	1.2–4.3	**0.018**
Maternal educational level	University	1	Reference	
	Below University	1.2	0.7–2.0	0.504
Parental overweight	No overweight/obese parents	1	Reference	
	Overweight/obese father	1.8	1.0–3.5	0.060
	Overweight/obese mother	2.7	1.3–5.9	**0.011**
	Both overweight/obese parents	3.4	1.7–7.1	**0.001**

OR, odds ratios; CI, confidence intervals; TV, television. * International Obesity Task Force body mass index cut-off values. † Waist circumference cut-off values for predicting risk factor clustering, as proposed by Bogalusa Heart Study. ‡ Logistic regression analysis was performed controlling for sex and age.

Children and adolescents that had a birth weight greater than 4000 g had two times higher odds of having a high-risk waist circumference, compared to those who had a birth weight between 2500 and 4000 g (*P *= 0.018). These results remained significant after additionally controlling for the different PA variables (*P *= 0.05, data not shown).

Parental overweight significantly increased the odds ratio of being overweight (OR from 2.0 to 3.4) and having a high-risk waist circumference (OR from 1.8 to 3.4, *P *values from 0.060 to 0.001). These results remained significant after additionally controlling for the different PA variables (*P *≤ 0.05, data not shown). No association was found between maternal educational level and overweight or high-risk waist circumference.

## Discussion

### Objectively measured physical activity

One of the major findings of this study was that those individuals with a low level of vigorous PA were more likely to be overweight, and more likely to have a high-risk waist circumference, compared to those who had a high level of vigorous PA. This finding is in accordance with those data reported by Gutin et al. and Ruiz et al. [16,17]. They observed that a high level of vigorous PA was associated with a lower adiposity in children and adolescents. Our data also suggest that being physically active, especially vigorously active, is associated with a lower "risk" of being overweight, independently of other important determinant factors, such as TV viewing, birth weight or parental overweight. Other authors have reported that when controlling the effect of dietary energy intake, PA was also the most important determinant of childhood obesity [40,41]. These findings call for interventions aiming to increase or maintain PA levels among young people. In this context, several school based-studies have succeeded in increasing PA levels [42,43].

The levels of vigorous PA associated with lower odds of being overweight and having a high-risk waist circumference were met at the third tertile. The sex and age-specific values for the third tertile were: ≥ 40 min/day and ≥ 25 min/day, for boys and girls, respectively, and ≥ 20 min/day and ≥ 15 min/day for adolescent boys and girls, respectively. Interestingly, data from the Swedish and Estonian EYHS-children aged 9–10 y, showed a significant difference between total body fat, as measured by skinfold thicknesses, of those who accumulated more than 40 min/day of vigorous PA and those who accumulated 10–18 min/day [17]. Of note is that although different adiposity indexes and statistical approach was used in that study, the figures reported are identical to those found in the current study in boys aged 9–10 y (>40 min/day). When accounting for sexual maturation status instead of age, the association between total PA and overweight was non-significant and vigorous PA still remained associated with both overweight and high-risk waist circumference.

Because the use of BMI for defining overweight/obesity in young people has been criticised, we additionally studied the associations discussed above using the high total fatness variable (derived from skinfold thicknesses) instead of the international BMI categories. Among the PA variables, only vigorous PA was associated with high total fatness. Data from a large UK project called "the Avon Longitudinal Study of Parents and Children", in which PA was measured by accelerometry and fatness by Dual Energy X-ray Absorciometry, also suggest that higher intensity PA may be more important than total PA in this matter [19]. Our findings, together with those reported by others, reinforce the thesis that high intensity PA may play a key role in the prevention of total and central childhood obesity. However, more data from longitudinal and randomized control trial are needed to support these results.

In accordance with other studies [19,44-46], our results showed higher levels of PA in boys than in girls and in children than adolescents. Nearly all the children involved in this study met the current PA recommendations (≥ 60 min of daily MVPA). Although this finding is in accordance with previously reported data from other European countries [44], the questions of whether the studied children are really active enough or if the PA recommendations are appropriate for this population remain unanswered. Andersen et al. (2006) reported that at least 90 min of daily MVPA is necessary to prevent a clustering of cardiovascular disease risk factors including excess of fatness in children and adolescents [47]. Our results support the hypothesis that 60 min or more of daily PA could be enough, if enough vigorous PA is accumulated during such period.

### Other determinant factors

Sedentary behaviour was associated with two-fold higher odds of having a high-risk waist circumference. Television viewing may increase the "risk" through both a reduction in energy expenditure or increased food intake [48,49]. Although there are potential benefits of viewing some TV shows, such as the promotion of positive aspects of social behaviour (eg, sharing, manners, and cooperation), many negative health effects can result [6]. In addition, longitudinal studies investigating the role of TV viewing on the development of obesity in youths suggest that decreased sedentary behaviour is protective against relative weight and fatness gains over childhood and adolescence [50,51]. In our study, when total PA or time spent in vigorous PA was taken into account, no association was found between TV viewing and high-risk waist circumference. This result suggests that the negative effect of spending more than 2 hours per day viewing TV on central fatness could be attenuated by an appropriate level of vigorous PA. This finding has important public health implications.

Another interesting finding was that those children and adolescents who had a high birth weight (>4000 g) were more likely to have a high-risk waist circumference, compared to those who had a normal birth weight (2500–4000 g). Associations between birth weight and overweight in childhood and adolescence have been well studied [8,52]. The main contribution of the current study in this regard is the association found between a high birth weight and a high-risk waist circumference. The U-shaped association reported by others [53] was not observed in our study. Our data also showed that this association is independent of the total PA, minutes spent in vigorous PA, TV viewing and parental overweight.

Parental overweight was an important determinant for overweight and high-risk waist circumference in the children and adolescents studied. Those subjects who had two overweight/obese parents showed three times higher odds of being overweight and having a high-risk waist circumference, compared to those whose parents were non-overweight. Similar findings have been previously reported in British children [11]. In addition, a longitudinal study reported that in either obese or non-obese children, parental obesity more than doubles the risk of adult obesity (OR = 5.0), particularly when both parents were obese [54]. Obesity in one or both parents probably influences the "risk" of obesity in their offspring because of shared genes and/or environmental factors within families. Our data also showed that this association is independent of total PA, time spent in vigorous PA, TV viewing and birth weight. To our knowledge, no study has previously examined the associations between parental overweight and waist circumference in children and adolescents.

### Limitations and strengths

It should be highlighted that the present cross-sectional study only provides suggestive evidence concerning causal relationships of PA and other determinant factors with overweight and high-risk waist circumference. Some limitations must be assumed in any study involving accelerometry, for instance, the fact that theaccelerometers do not compensate for the relative increase in energy expenditure with increasing in body size. Nevertheless, accelerometry is nowadays a reference method in epidemiological studies. The fact that a relatively large sample of children and adolescents were assessed by means of accelerometry in relation to total and central obesity, taking into account several well known determinant factors, is a notable strength of this study.

## Conclusion

The results suggest that low levels of total PA and mainly vigorous PA may play an important role in the development of overweight and excess of central adiposity in children and adolescents, independently of important determinant factors, such as TV viewing and birth weight. In addition vigorous PA predicted the "risk" of being overweight independent of parental overweight, which also showed a strong association with overweight and high-risk waist circumference in children and adolescents. Spending more than 2 h/day in TV viewing seems to be related to having a high-risk waist circumference. However, the data indicate that its effect on central fat deposition could be attenuated if enough vigorous PA is accumulated. The lack of sex and age interactions observed suggest that the findings and conclusions raised in this study are consistent for boys and girls and for children and adolescents. Intervention studies are needed to confirm in what way and to what extent changes in lifestyle may influence on these relationships.

## Abbreviations

BMI, body mass index

CI, confidence intervals

IOTF, International Obesity Task Force

MVPA, moderate to vigorous physical activity

OR, odds ratio

PA, physical activity

## Competing interests

The author(s) declare that they have no competing interests.

## Authors' contributions

FBO has been the main responsible for analysis and interpretation of data and drafting the manuscript. JRR has been involved in revising the manuscript critically for important intellectual content. MS was the main responsible for the study conception and design, acquisition of funding, collecting of data, and has revised the manuscript critically. All the authors have given final approval of the current version to be published.
